# Genetic Risk for Depression Associates with Circulating Immunoregulatory Natural Killer Cells Independent of BMI: An Exploratory Immunophenotyping Study

**DOI:** 10.3390/cells15131179

**Published:** 2026-06-29

**Authors:** Aikaterini Fyka, Dimitra Anastasopoulou, Marina Livadara, Aristides G. Eliopoulos, Kalliopi Gkouskou

**Affiliations:** Department of Biology, School of Medicine, National and Kapodistrian University of Athens, 11527 Athens, Greece; katerina44f@gmail.com (A.F.); diman1321@gmail.com (D.A.); mlivadara@gmail.com (M.L.)

**Keywords:** NK cells, depression, genetic risk score, obesity, immunophenotyping, BMI

## Abstract

**Highlights:**

**Main findings**

- A depression genetic risk score (GRS) is correlated positively with BMI and CRP across the obesity spectrum, consistent with the shared genetic architecture between depression and inflammatory–metabolic traits.
- Depression-GRS is associated with CD56^bright^CD16^−^ NK cells independently of BMI, age, sex, and physical activity.

**Implications of the main findings**

- Genetic predisposition to depression is constitutively reflected in the peripheral immunophenotype, implicating the CD56^bright^CD16^−^ NK cell compartment in depression neuroimmune biology.
- The potential link between elevated CD56^bright^CD16^−^ NK cell proportions, IFN-γ production, and kynurenine pathway dysregulation in depression susceptibility warrants direct functional investigation.

**Abstract:**

Depressive disorders and obesity are highly comorbid conditions sharing genetic, metabolic, and immunological substrates. In a cross-sectional analysis of 53 participants across the obesity spectrum (lean *n* = 12; overweight *n* = 9; obese *n* = 32), a depression genetic risk score (d-GRS) correlated positively with BMI (*ρ* = 0.379, *p* = 0.005) and with serum CRP (*ρ* = 0.322, *p* = 0.031), consistent with the known genetic coarchitecture between depression and inflammatory traits. The d-GRS was tested against 116 flow-cytometry-derived immune parameters using Spearman rank correlation. The most consistent immune association at nominal significance (*p* < 0.05, uncorrected) involved the immunoregulatory CD56^bright^CD16^−^ natural killer (NK) cell subset across two independent gate representations (*ρ* = 0.444, *p* = 0.004), remaining significant after sequential adjustment for BMI, sex, age, and physical activity (adjusted *ρ* range: 0.439–0.469), with no equivalent association for a genetically independent obesity GRS. In silico analysis of d-GRS SNP-tagged genes identified several with documented roles in NK cell trafficking, activation, and cytokine production, providing a putative mechanistic basis for this association. These findings nominate the CD56^bright^CD16^−^ NK cell subset as a candidate immunological link between depression genetic susceptibility and neuroimmune mechanisms, warranting independent replication and functional characterisation in prospective cohorts.

## 1. Introduction

Major depressive disorder (MDD) and obesity are leading causes of disability worldwide and frequently co-occur, with observational studies consistently reporting a bidirectional relationship between the two conditions. Beyond behavioural and environmental pathways, genome-wide association studies have begun to uncover shared genetic architecture. Bahrami et al. [1] identified 32 genetic loci shared between major depression and BMI in a dataset of over 1.38 million participants, and subsequent analyses demonstrated that BMI increases the risk of MDD in individuals with high polygenic burden for depression [2].

The immune system represents a plausible biological interface between depression genetics and metabolic phenotype. Evidence from a large meta-analysis [3] indicates that MDD is associated with elevated counts of NK cells, neutrophils, monocytes, and B cells in peripheral blood. However, whether these immune alterations reflect constitutional genetic predisposition or are secondary to the clinical state, pharmacotherapy, or behavioural consequences of active depressive illness remains unresolved. Indeed, case–control designs comparing clinically diagnosed individuals with healthy controls are inherently susceptible to such state-dependent confounding, limiting their ability to identify trait-like immune correlates of genetic susceptibility.

Among NK cell subsets, the CD56^bright^CD16^−^ is of particular interest given its immunoregulatory function through the production of IFN-γ, TNF, and IL-10 [4]. As a prime peripheral source of IFN-γ among NK cell subsets, CD56^bright^CD16^−^ cells are positioned to activate the kynurenine pathway, diverting tryptophan metabolism toward neuroactive metabolites implicated in depression pathophysiology [5]. This is particularly relevant in the present cohort, given that the proportion of CD56^bright^CD16^−^ cells in obesity is inconsistent across studies, with some reporting increases in obesity [6] and others finding no significant difference compared to normal-weight individuals [7]. Importantly, Lynall et al. demonstrated that genetic risk variants for depression are enriched at epigenetically active regulatory sites in lymphoid cells, including NK cells, but not in myeloid cells [8], providing a direct genetic rationale for investigating this subset in the context of depression polygenic burden.

Genetic risk scores (GRSs) aggregate the cumulative burden of common genetic variants associated with a trait and can be used to probe biological associations independently of clinical diagnosis and environmental confounders [2,9]. Unlike clinical diagnosis which reflects a phenotypic threshold shaped by current disease state and treatment, GRSs remain invariant across clinical states and behavioural factors, making them well-suited to identify trait-like biological correlates of genetic susceptibility. Despite the growing evidence linking depression genetics to immune dysregulation, it remains unclear whether polygenic burden for depression associates with specific circulating immune cell subsets or with metabolic parameters across the obesity spectrum. To this end, we examined associations between a depression GRS (d-GRS) [2] and a comprehensive panel of 116 flow-cytometry-derived immune parameters, selected biochemical indices, and BMI in a well-characterised cohort spanning lean to obese. An obesity GRS (Ob-GRS) [10] was additionally included as a genetically independent comparator to assess the specificity of any observed associations for depression-related genetic architecture.

## 2. Materials and Methods

### 2.1. Participants

This was a cross-sectional study of 53 adult participants (35 females, 18 males; mean age 38.3 ± 11.3 years, range 18–66) recruited from 2017–2019, stratified into three groups according to BMI: lean (BMI < 25 kg/m^2^, *n* = 12), overweight (BMI 25–29.9 kg/m^2^, *n* = 9), and obesity (BMI ≥ 30 kg/m^2^, *n* = 32). Participant characteristics are summarised in Table 1. Participants with a known inflammatory condition, autoimmune disease, or active infection were excluded. No participant was receiving immunomodulatory, antidepressant, or psychotropic medication at the time of assessment. Among participants with obesity, three were receiving antihypertensive medication, three were receiving statins, and two were receiving thyroid medication. None of these medications is known to directly alter circulating NK cell subset proportions. Clinical depression status and psychiatric history were not formally assessed; participants were not recruited on the basis of depressive symptomatology. Valid n per individual analysis ranged from 31 to 53 due to pairwise deletion of missing values; the 12 participants with missing immunophenotyping data had insufficient PBMC yield at the time of processing and were excluded from NK cell analyses only. Ethical approval was obtained from the NKUA Medical School Ethics Committee, reference number 1718034127. All participants provided written informed consent.

### 2.2. Depression and Obesity GRSs

The d-GRS used herein is based on 30 depression-associated SNPs identified in Anguita-Ruiz et al. [2], selected from an initial pool of 56 candidate variants from candidate gene association studies and GWAS meta-analyses [2]. In our implementation we applied a weighted scoring approach in which each risk allele was multiplied by the corresponding β coefficient from the logistic regression model reported in Anguita-Ruiz et al. [2], thereby accounting for the differential effect size of each variant. Individual weighted scores were subsequently compared to a reference population of approximately 624 Greek individuals to derive percentile-based classifications of low, moderate, and high genetic risk. The GRS accounts for approximately 4.17% of the variance in depression status [2], and SNP minor allele frequencies were verified to be ≥0.05 and comparable to those reported for Southern European populations, supporting its applicability to the present cohort. Full SNP details are provided in Supplementary Material of reference [2]. An Ob-GRS, constructed from 32 SNPs associated with BMI [10], was included as a genetically independent specificity comparator for the NK cell analyses; the d-GRS and Ob-GRS were confirmed to be mutually independent (Spearman *ρ* = −0.078, *p* = 0.582, *n* = 52). DNA isolation and genotyping, along with application of Ob-GRS in a Greek population, were previously described [9]. Both GRSs were treated as continuous variables in all analyses.

### 2.3. Immunophenotyping

Peripheral blood mononuclear cells (PBMCs) were isolated from fasting venous blood samples by density gradient centrifugation and analysed by multiparameter flow cytometry as previously described [11,12]. A total of 116 immune parameters spanning T cell subsets (CD3^+^, CD4^+^, CD8^+^, naïve, effector memory, central memory, TEMRA), B cells (CD19^+^), NK cell subsets (CD56^bright^CD16^−^, CD56^dim^CD16^+^), monocyte subsets, neutrophils, and regulatory T cells were quantified as percentages of parent gates. Parameters were expressed relative to multiple parent populations (SS/FS, DAPI^−^, CD45^+^, and lineage-specific gates; Table S1). All samples were processed within two hours of venepuncture under standardised conditions.

### 2.4. Biochemical and Haematological Analyses

Standard fasting biochemical analyses were performed on venous blood samples collected at the same visit as immunophenotyping. Analyses included serum glucose, lipid profile (total cholesterol, triglycerides, HDL, LDL), liver enzymes (ALT, γ-GT), renal function markers (creatinine, uric acid), electrolytes, ferritin, vitamin B12, folic acid, magnesium, albumin, TSH, vitamin D3, and high-sensitivity C-reactive protein (hsCRP). Full blood count included haemoglobin, haematocrit, MCV, MCH, MCHC, RDW, and WBC.

### 2.5. Statistical Analysis

Spearman rank correlation was used to test associations between the d-GRS and 142 continuous variables (116 immune parameters, 25 biochemical/haematological parameters, and BMI). Benjamini–Hochberg false discovery rate (FDR) correction was applied across all 142 tests; no association survived correction (all q > 0.05) and all findings are therefore reported at the nominal significance level and should be regarded as hypothesis-generating. To assess independence of the CD56^bright^CD16^−^ NK cell association from adiposity at the genetic level, Spearman correlations were additionally computed between the Ob-GRS and CD56^bright^CD16^−^ NK cell proportions across three independent gate representations; these analyses were performed as specificity tests and were not included in the FDR correction family. To address potential confounding by demographic and lifestyle factors, partial Spearman correlations were computed between the d-GRS and CD56^bright^CD16^−^ NK cell proportions after sequential adjustment for BMI, sex, age, and physical activity level using the residuals method on ranked data; an additional model further adjusted for hsCRP. A Kruskal–Wallis test with Bonferroni-corrected post hoc Mann–Whitney U tests was used to compare CD56^bright^CD16^−^ NK cell proportions across weight groups. Confidence intervals for Spearman correlations were calculated using Fisher’s z-transformation. Power was estimated using Fisher’s z-transformation with the present sample provided >80% power to detect the observed primary effect sizes (*ρ* = 0.379–0.444) at uncorrected α = 0.05, whereas approximately 90 complete paired observations would be required to achieve 80% power at a conservative FDR-corrected threshold (α = 0.05/142). All analyses were conducted in Python 3 (scipy, statsmodels).

## 3. Results

### 3.1. Participant Characteristics

Participant characteristics stratified by weight group are presented in Table 1. Age did not differ significantly across weight groups (Kruskal–Wallis *p* = 0.595). Physical activity was significantly lower in the obese group compared to lean participants (*p* = 0.025), and CRP was significantly higher in the obese group, consistent with obesity-related low-grade inflammation (*p* = 0.035). Haemoglobin and triglyceride levels differed significantly across groups (*p* = 0.002 and *p* = 0.001 respectively). The d-GRS differed significantly across weight groups (H = 14.19, *p* < 0.001), driven primarily by higher scores in the obese group.

### 3.2. Depression-GRS Associates with BMI and Biochemical Indices

**Figure 1:**
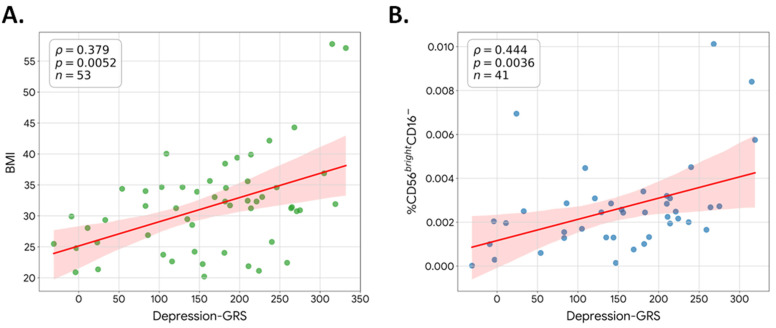
Association of d-GRS with BMI and CD56^bright^CD16^−^ NK cells. (**A**) Spearman correlation between d-GRS and BMI. (**B**) Spearman correlation between d-GRS and %CD56^bright^CD16^−^ NK cells. In both (**A**,**B**), each point represents one participant. The lines represent the rank-based trends. Spearman’s rho (*ρ*) and corresponding *p*-values are displayed.

A positive association was found between the d-GRS and BMI across the full cohort (*ρ* = 0.379, 95%CI [0.121–0.589], *p* = 0.005, *n* = 53), indicating that higher polygenic burden for depression is associated with greater adiposity (Figure 1A). Sex was not associated with either the d-GRS (*ρ* = 0.211, *p* = 0.129) or BMI (*ρ* = −0.089, *p* = 0.528), confirming that it does not confound the primary associations reported here.

Among 25 biochemical and haematological indices tested, three reached nominal significance, namely haemoglobin (*ρ* = 0.400, 95%CI [0.137–0.610], *p* = 0.004, *n* = 50), triglycerides (*ρ* = 0.351, 95%CI [0.077–0.575], *p* = 0.013, *n* = 49) and CRP (*ρ* = 0.322, 95%CI [0.032–0.563], *p* = 0.031, *n* = 45) (Table 2). Serum glucose showed a trend in the same direction that did not reach nominal significance (*ρ* = 0.246, *p* = 0.085). No biochemical association survived Benjamini–Hochberg FDR correction.

### 3.3. Association of d-GRS with Immune Parameters

Spearman rank correlations were computed between the d-GRS and 116 PBMC-derived immune parameters, encompassing T cell subsets (naïve, central memory, effector memory, TEMRA), B cells, NK cell subsets (CD56^bright^CD16^−^, CD56^dim^CD16^+^), monocyte subsets, neutrophils, and regulatory T cells, expressed relative to multiple parent gates (Table S1). Twelve parameters reached nominal significance (*p* < 0.05; Table 3); none survived Benjamini–Hochberg FDR correction, and all associations are thus hypothesis-generating.

The most consistent signal involved the immunoregulatory CD56^bright^CD16^−^ NK cell subset, which showed nominally significant positive correlations with the d-GRS across two independent parent gate representations, the percentage of viable DAPI^−^ cells (*ρ* = 0.444, 95%CI [0.158–0.661], *p* = 0.004, *n* = 41) and the percentage of CD45^+^ leukocytes (*ρ* = 0.342, 95%CI [0.038–0.588], *p* = 0.029, *n* = 41; Figure 1B; Table 3). Total NK cells (%CD56^+^) also reached nominal significance in two gates. Additional nominally significant associations were observed with antigen-experienced %CD45RA^−^CD45RO^+^ memory T cells (two gates, *ρ* = 0.331 and *ρ* = 0.282), effector memory %CD45RO^+^CCR7^−^ (*ρ* = 0.308), central memory %CD45RO^+^CXCR3^+^ (*ρ* = 0.275) and naïve %CD45RA^+^CCR7^+^ cells (*ρ* = 0.274, *p* = 0.050). A nominally significant inverse association was also observed with central memory non-NK CD56^−^ cells (*ρ* = −0.353, *p* = 0.024). Overall, the CD56^bright^CD16^−^ subset emerged as the most biologically coherent cluster given its representation across independent gates and its distinct immunoregulatory function within the NK cell lineage.

### 3.4. The Association of d-GRS with the Proportion of CD56^bright^CD16^−^ NK Cells Is Independent of BMI, Lifestyle Factors, and Systemic Inflammation

To systematically assess whether the CD56^bright^CD16^−^ NK cell association with the d-GRS could be attributed to adiposity or other potential confounders, we conducted analyses at phenotypic, statistical, and genetic levels.

At the phenotypic level, %CD56^bright^CD16^−^ cells showed no correlation with BMI (*ρ* = 0.089, 95%CI [−0.225–0.386], *p* = 0.580, *n* = 41) and did not differ across normal weight, overweight, and obese groups (Kruskal–Wallis H = 0.46, *p* = 0.793), with all Bonferroni-corrected post hoc pairwise comparisons non-significant. Neither age (*ρ* = 0.070, *p* = 0.669), physical activity (*ρ* = −0.101, *p* = 0.540), nor CRP (*ρ* = 0.103, *p* = 0.545, *n* = 37) was associated with CD56^bright^CD16^−^ proportions, confirming that none represents an independent confounder of the primary association.

At the statistical level, Spearman correlations were computed after sequential adjustment for potential confounders (Table 4). The association was remarkably stable across models 1–5 (Table 4), remaining nominally significant and virtually unchanged in magnitude following adjustment for BMI, sex, age, and physical activity (*ρ* range: 0.439–0.469; all *p* ≤ 0.007; *n* range: 38–41). In the fully adjusted model additionally incorporating CRP (model 6), the association attenuated to non-significance (*ρ* = 0.263, 95%CI [−0.082–0.552], *p* = 0.167, *n* = 34). This attenuation could be attributed to two factors: first, the reduction in sample size from *n* = 38 to *n* = 34 due to missing CRP values; and second, partial collinearity between d-GRS and CRP (*ρ* = 0.322, *p* = 0.031) which causes CRP to absorb a proportion of the variance associated with the d-GRS without representing genuine confounding, given that CRP itself showed no association with %CD56^bright^CD16^−^ (*ρ* = 0.103, *p* = 0.545).

To address adiposity confounding at the genetic level, we tested an Ob-GRS, a genetically independent score constructed from SNPs associated with BMI [9,10], against CD56^bright^CD16^−^ NK cell proportions in the same participants. The Ob-GRS showed no association with this subset across all three parent gate representations (to DAPI^−^: *ρ* = −0.114, *p* = 0.482; to CD45^+^: *ρ* = −0.130, *p* = 0.426; to CD56^+^: *ρ* = −0.053, *p* = 0.746; *n* = 40), with effect sizes near zero and directionally opposite to those observed for the d-GRS. Together, the aforementioned phenotypic, statistical, and genetic lines of evidence consistently indicate that the CD56^bright^CD16^−^ NK cell association with the d-GRS reflects depression-related genetic architecture rather than a secondary consequence of adiposity, lifestyle factors or obesity-associated immune dysregulation.

Sex was not associated with CD56^bright^CD16^−^ NK cell proportions (*ρ* = −0.031, *p* = 0.847, *n* = 41), confirming it does not confound the primary association. In exploratory sex-stratified analyses, the d-GRS to CD56^bright^CD16^−^ association appeared stronger in males (*ρ* = 0.731, 95%CI 0.301–0.914, *p* = 0.005, *n* = 13) than in females (*ρ* = 0.335, 95%CI −0.043–0.630, *p* = 0.081, *n* = 28), suggesting possible sex-dependent modification of the association. However, the difference between the two correlation coefficients was not statistically significant (Fisher’s z test, *p* = 0.120), and the male subgroup (*n* = 13) was insufficient for reliable inference; this observation warrants investigation in larger sex-balanced cohorts.

## 4. Discussion

The central finding of this study is the association between the d-GRS and the immunoregulatory CD56^bright^CD16^−^ NK cell subset, detectable in a cohort not selected for clinical depression and independent of adiposity across phenotypic, statistical, and genetic lines of evidence. Elevated NK cell counts have been reported in MDD predominantly in case–control studies comparing clinically diagnosed individuals with healthy controls [3]. Such designs are susceptible to state-dependent confounding, as immune parameters measured during active depressive illness may reflect the neurobiological sequelae of chronic stress, pharmacotherapy, disrupted sleep, and/or comorbid inflammatory conditions rather than constitutional genetic predisposition [13]. This is supported by evidence that several lymphocyte subpopulations normalise following pharmacotherapy and clinical improvement in MDD, whereas NK cell parameters remain unchanged [14], suggesting that distinct immune compartments may index different biological dimensions of depression, some state-dependent, others potentially trait-like.

GRSs aggregate the additive effects of common genetic variants, providing a measure of genetic susceptibility that is independent of current clinical state, pharmacological treatment, or behavioural factors [2]. The detection of a d-GRS-associated immunophenotypic signal in individuals not selected for clinical depression suggests that variation in the innate lymphoid compartment may reflect part of the biological underpinnings of genetic susceptibility to depression, operating upstream of clinical manifestations of disease. This interpretation is consistent with the diathesis–stress model of depression, in which polygenic predisposition interacts with environmental exposures over the lifespan to determine whether and when clinical threshold criteria are met [15].

The biological plausibility of this association is supported by converging genomic evidence. Lynall et al. demonstrated that depression risk variants are significantly enriched at epigenetically active regulatory sites in lymphoid cells, including NK cells, but not in myeloid cells [8]. This cell-type specificity provides a direct mechanistic rationale for a d-GRS–NK cell relationship that may not be detectable using clinical diagnosis as the index of depression-related biology, given that diagnosis captures the phenotypic endpoint rather than the upstream genetic regulatory architecture.

The CD56^bright^CD16^−^ subset is of particular mechanistic relevance as these cells represent the immunoregulatory, cytokine-producing arm of the NK lineage and constitute the predominant NK population within secondary lymphoid tissues, the sites of highest gene-regulatory activity [4]. Furthermore, genetic susceptibility to depression is associated with dysregulation of the HPA axis, resulting in sustained elevation of circulating cortisol [16]. Given the established sensitivity of CD56^bright^CD16^−^ cells to glucocorticoid-mediated modulation [17], this endocrine dysregulation represents a plausible constitutional mechanism linking depression-related polygenic burden to the NK cell compartment.

Our in silico analysis of genes tagged by SNPs in the d-GRS identified several with documented roles in NK cell biology, providing a plausible gene-level mechanistic basis for the observed association (Table S2). Specifically, *ITGB1* encoding integrin β1 mediates NK cell adhesion and tissue trafficking [18] and *RORA*, a nuclear receptor regulating both circadian rhythm and innate lymphoid cell development, controls cytokine production in innate lymphoid populations [19]. *HTR1A*, encoding the serotonin 5-HT_1_A receptor, has been shown to mediate serotonergic regulation of NK cell cytotoxicity and proliferation in the context of monocyte–NK cell interactions [20]. COMT degrades catecholamines, including dopamine, which regulates NK cell cytotoxicity via dopamine receptors expressed on NK cells [17]. Additionally, *CRHR1*, *CRHR2*, and *CRHBP* encode components of CRH signaling whose dysregulation is associated with altered glucocorticoid output [16] known to suppress NK cell function [17]. While these observations are post hoc and the SNPs were selected for their association with depression rather than NK cell function, the convergence of depression-associated variants on pathways governing NK cell trafficking, activation, and cytokine production provides a putative gene-level framework for the GRS–NK cell association reported here.

What could be the potential relevance of this association to depression pathophysiology? Whereas this remains to be established experimentally, it is tempting to speculate that, as a primary peripheral source of IFN-γ among NK cell subsets [4,21], CD56^bright^CD16^−^ NK cells could activate indoleamine 2,3-dioxygenase (IDO), the rate-limiting enzyme of the kynurenine pathway. IDO-mediated tryptophan catabolism diverts substrate away from serotonin synthesis toward the production of neuroactive and potentially neurotoxic kynurenine metabolites implicated in the pathophysiology of depression [5]. Elevated IFN-γ is among the most consistently reported immune findings in MDD, and the IFN-γ–IDO–kynurenine axis represents one of the most mechanistically characterised pathways linking peripheral immune activation to neurobiology of depression [22]. Additionally, CD56^bright^CD16^−^ cells express the chemokine receptors CXCR3 and CCR7 that confer capacity for migration toward sites of inflammation and into secondary lymphoid tissues [23], raising the possibility that their increased peripheral representation reflects immune-regulatory processes with potential relevance to depression.

Genome-wide analyses have identified shared genetic loci between depressive disorders and BMI [1]. The d-GRS correlated positively with BMI in the present cohort (*ρ* = 0.379, *p* = 0.005), consistent with the established shared genetic architecture between depression and adiposity-related traits [1] and with evidence that depression-related genetic burden interacts with metabolic phenotype to amplify disease risk [2]. This observation, together with the known influence of adipose tissue on NK cell biology [24], led us to hypothesise that the d-GRS–NK cell association could be mediated through obesity-derived cytokines and metabolic inflammation. Four converging lines of evidence argue against an adiposity-mediated interpretation. First, the %CD56^bright^CD16^−^ did not correlate with BMI (*ρ* = 0.089, *p* = 0.580) and proportions were uniform across normal weight, overweight, and obesity groups (Kruskal–Wallis H = 0.46, *p* = 0.793), consistent with a previous report [7]. Second, the d-GRS to CD56^bright^CD16^−^ association was virtually unchanged after accounting for BMI (unadjusted *ρ* = 0.444, *p* = 0.004; BMI-adjusted *ρ* = 0.439, *p* = 0.005) and remained stable across sequential models adjusting for BMI, sex, age, and physical activity (*ρ* range: 0.439–0.469; all *p* ≤ 0.007; Table 4). Third, an Ob-GRS that is genetically independent of the d-GRS (*ρ* = −0.078, *p* = 0.582) showed no association with CD56^bright^CD16^−^ cells across three independent gate representations (*ρ* range: −0.114 to −0.053; all *p* > 0.42). The fact that the Ob-GRS and d-GRS showed directionally opposite associations with CD56^bright^CD16^−^ cells further argues against a generic polygenic effect on this subset. Fourth, neither physical activity nor CRP was independently associated with CD56^bright^CD16^−^ proportions (*ρ* = −0.101, *p* = 0.540 and *ρ* = 0.103, *p* = 0.545 respectively), further dissociating the NK cell signal from obesity-related lifestyle and inflammatory factors. Of note, the d-GRS was nominally associated with CRP (*ρ* = 0.322, *p* = 0.031), consistent with the known link between depression genetic susceptibility and systemic inflammation [25,26]. However, given the absence of a CRP–CD56^bright^ NK association, this reflects a biological relationship between depression polygenic burden and inflammation that is independent of the NK cell compartment.

Several limitations warrant consideration. The modest sample size is the primary constraint. As no association survived Benjamini–Hochberg FDR correction across 142 tests, all findings must be regarded as hypothesis-generating, requiring independent replication before definitive conclusions can be drawn. The multiple-testing burden in exploratory immunophenotyping studies of this scale inevitably carries a non-trivial false-positive risk: at uncorrected α = 0.05 across 142 tests, approximately seven false-positive associations would be expected by chance alone, and the 12 nominally significant associations reported here cannot be individually validated without correction. Power analysis indicated adequate power (>80%) to detect the observed effect sizes at uncorrected α = 0.05; however, approximately *n* ≈ 90 complete paired observations would be required for confirmation at a conservative FDR-corrected threshold. Reproducibility of the primary finding—the d-GRS to CD56^bright^CD16^−^ NK cell association—is supported by its consistency across two independent gate representations, its stability across five sequential multivariable adjustment models, and its absence for a genetically independent Ob-GRS tested in the same participants. Nonetheless, independent replication in an adequately powered cohort remains essential.

Clinical depression status and depressive symptom severity were not independently assessed, precluding evaluation of whether the association is modified by current or lifetime depressive disorder. Whereas self-reported depressive disorder or psychiatric illness was negative for this cohort, future studies should address the d-GRS–NK cell association through structured psychiatric interviews.

The absence of functional NK cell data, including IFN-γ secretion assays, cytotoxicity measurements, and IDO activity, limits interpretation of the proportional CD56^bright^CD16^−^ findings reported here and prevents direct testing of the kynurenine pathway hypothesis. Additionally, data on smoking status, alcohol consumption, and dietary habits were not available for this cohort; as these lifestyle factors are known to influence circulating NK cell populations, their absence represents a limitation that should be addressed in future studies. Finally, replication in larger, clinically characterised cohorts incorporating depressive symptom assessment, endocrine profiling, functional NK cell assays, and longitudinal follow-up is required to determine whether this immunogenetic signal predicts depressive onset, persistence, or treatment response.

## 5. Conclusions

In summary, this exploratory immunophenotyping study provides initial hypothesis-generating evidence that depression-related polygenic burden is associated with elevated proportions of circulating CD56^bright^CD16^−^ NK cells. Although none of the associations survived Benjamini–Hochberg FDR correction and independent replication is required before biological conclusions can be drawn, the nominal association was consistent across phenotypic, statistical, genetic, and genomic lines of evidence and independent of adiposity across four distinct analytical approaches. Given the role of CD56^bright^CD16^−^ cells as primary IFN-γ producers and their potential to activate the kynurenine pathway, these findings raise the possibility that the immunoregulatory NK cell compartment may represent a candidate peripheral link between depression genetic susceptibility and neuroimmune mechanisms, warranting functional characterisation and independent replication in larger prospective cohorts.

## Figures and Tables

**Table 1:** Participant characteristics by weight group: Values are median (IQR) unless otherwise stated; sex is presented as *n* (%). The *p*-values are from Kruskal–Wallis test across three groups. PA = physical activity; CRP = C-reactive protein; d-GRS = Depression Genetic Risk Score. %CD56^bright^ NK cells expressed as ×10^−3^ of DAPI^−^ cells.

Variable	Lean (*n* = 12)	Overweight (*n* = 9)	Obese (*n* = 32)	Total (*n* = 53)	*p*
Age (years)	33.5 (25.0–48.2)	36.0 (31.8–42.8)	39.5 (31.8–45.2)	37.5 (29.0–45.2)	0.595
Females, *n* (%)	5 (42%)	7 (78%)	23 (72%)	35 (66%)	—
BMI (kg/m^2^)	22.3 (21.3–23.8)	28.0 (25.8–29.3)	34.2 (31.9–37.3)	31.4 (25.7–34.6)	<0.001
Physical activity (h/week)	3.0 (3.0–4.0)	2.0 (2.0–3.0)	1.5 (0.0–3.0)	2.0 (0.0–3.0)	0.025
CRP (mg/dL)	0.050 (0.030–0.400)	0.120 (0.040–0.400)	0.500 (0.150–1.195)	0.400 (0.080–0.800)	0.035
Haemoglobin (g/dL)	14.9 (13.4–15.1)	12.6 (12.2–12.8)	13.4 (13.0–14.6)	13.4 (12.9–14.8)	0.002
Triglycerides (mg/dL)	74 (62–87)	70 (64–77)	109 (83–124)	87 (72–115)	0.001
d-GRS	149 (85–188)	33 (11–135)	210 (159–264)	181 (105–228)	<0.001
%CD56^bright^CD16^−^ NK cells (×10^−3^)	2.167 (1.678–2.507)	2.233 (1.229–2.858)	2.468 (1.381–3.087)	2.435 (1.324–2.864)	0.793

**Table 2:** d-GRS associations with biochemical and haematological indices.

Variable	*n*	*ρ*	*p*-Value
Serum Glucose (mg/dL)	49	0.240	0.097
Creatinine (mg/dL)	48	0.113	0.446
Serum Uric Acid (mg/dL)	49	0.187	0.197
Potassium (mmol/L)	45	0.162	0.287
Sodium (mmol/L)	45	−0.087	0.570
Ferritin (ng/mL)	47	0.224	0.130
Vitamin B12 (pg/mL)	48	0.056	0.707
Folic Acid (ng/mL)	43	0.043	0.784
Magnesium (mg/dL)	44	−0.049	0.752
Cholesterol (mg/dL)	49	0.204	0.160
Triglycerides (mg/dL)	49	0.351	0.013
SGPT/ALT (U/L)	48	0.192	0.191
γ-GT (U/L)	49	0.141	0.334
Albumin (g/dL)	42	−0.187	0.236
TSH (mIU/L)	47	0.079	0.600
Vitamin D3 (ng/mL)	37	−0.295	0.076
CRP (mg/dL)	45	0.322	0.031
RBC (×10^6^/μL)	48	0.191	0.194
Haemoglobin (HGB) (g/dL)	50	0.400	0.004
Haematocrit (HCT) (%)	50	0.254	0.075
MCV (fL)	49	−0.141	0.335
MCH (pg)	49	0.062	0.674
MCHC (g/dL)	49	0.259	0.073
RDW	45	0.033	0.832
WBC (κ./μL)	49	0.051	0.727

**Table 3:** Associations of d-GRS with immune cell subpopulations.

Immune Population	Parent Population	*ρ*	*p*-Value	*n*
%CD56^bright^CD16^−^	DAPI^−^	0.444	0.0036	41
%CD56^bright^CD16^−^	CD45^+^	0.342	0.0286	41
%CD45RA^−^ CD45RO^+^	DAPI^−^	0.331	0.0164	52
Central memory CD56^−^	CD45^+^	−0.353	0.0235	41
Effector memory CD45RO^+^ CCR7^−^	DAPI^−^	0.308	0.0263	52
%CD56^+^ NK cells	DAPI^−^	0.321	0.0405	41
%CD56^+^ NK cells	CD45^+^	0.320	0.0411	41
Naïve %CD45RA^+^ CCR7^+^	DAPI^−^	0.274	0.050	52
Central memory %CD45RO^+^ CXCR3^+^	DAPI^−^	0.275	0.049	52

**Table 4:** Sequential partial Spearman correlations between d-GRS and %CD56^bright^CD16^−^ NK cells. PA = physical activity; CRP = C-reactive protein.

Model	*n*	*ρ*	95% CI	*p*
Unadjusted	41	0.444	0.158–0.661	0.004
+ BMI	41	0.439	0.151–0.658	0.005
+ BMI, sex	41	0.469	0.188–0.679	0.003
+ BMI, sex, age	40	0.461	0.175–0.676	0.004
+ BMI, sex, age, PA	38	0.454	0.157–0.676	0.007
+ BMI, sex, age, PA, CRP	34	0.263	−0.082–0.552	0.167

## Data Availability

The original contributions presented in this study are included in the article/Supplementary Materials. Further inquiries can be directed to the corresponding authors.

## Supplementary Materials

The following supporting information can be downloaded at: https://www.mdpi.com/article/10.3390/cells15131179/s1, Table S1: Descriptive statistics and Spearman correlations with the Depression-GRS for all 116 flow-cytometry-derived immune parameters; Table S2: Biological pathway categories identified among genes represented in the Depression-GRS.

## Abbreviations

The following abbreviations are used in this manuscript:

HPA	Hypothalamic-Pituitary-Adrenal
GRS	Genetic Risk Score
NK	Natural Killer (cells)
RDW	Red Cell Distribution Width (%)
WBC	White Blood Cell

## References

[1] Bahrami S., Steen N.E., Shadrin A., O’Connell K., Frei O., Bettella F., Wirgenes K.V., Krull F., Fan C.C., Dale A.M. (2020). Shared Genetic Loci Between Body Mass Index and Major Psychiatric Disorders: A Genome-wide Association Study. JAMA Psychiatry.

[2] Anguita-Ruiz A., Zarza-Rebollo J.A., Perez-Gutierrez A.M., Molina E., Gutierrez B., Bellon J.A., Moreno-Peral P., Conejo-Cerón S., Aiarzagüena J.M., Ballesta-Rodríguez M.I. (2022). Body mass index interacts with a genetic-risk score for depression increasing the risk of the disease in high-susceptibility individuals. Transl. Psychiatry.

[3] Foley E.M., Parkinson J.T., Mitchell R.E., Turner L., Khandaker G.M. (2023). Peripheral blood cellular immunophenotype in depression: A systematic review and meta-analysis. Mol. Psychiatry.

[4] Poli A., Michel T., Theresine M., Andres E., Hentges F., Zimmer J. (2009). CD56^bright^ natural killer (NK) cells: An important NK cell subset. Immunology.

[5] Savitz J. (2020). The kynurenine pathway: A finger in every pie. Mol. Psychiatry.

[6] Bahr I., Jahn J., Zipprich A., Pahlow I., Spielmann J., Kielstein H. (2018). Impaired natural killer cell subset phenotypes in human obesity. Immunol. Res..

[7] Laue T., Wrann C.D., Hoffmann-Castendiek B., Pietsch D., Hubner L., Kielstein H. (2015). Altered NK cell function in obese healthy humans. BMC Obes..

[8] Lynall M.E., Soskic B., Hayhurst J., Schwartzentruber J., Levey D.F., Pathak G.A., Polimanti R., Gelernter J., Stein M.B., Trynka G. (2022). Genetic variants associated with psychiatric disorders are enriched at epigenetically active sites in lymphoid cells. Nat. Commun..

[9] Gkouskou K.G., Georgiopoulos G., Vlastos I., Lazou E., Chaniotis D., Papaioannou T.G., Mantzoros C.S., Sanoudou D., Eliopoulos A.G. (2022). CYP1A2 polymorphisms modify the association of habitual coffee consumption with appetite, macronutrient intake, and body mass index: Results from an observational cohort and a cross-over randomized study. Int. J. Obes..

[10] Qi Q., Chu A.Y., Kang J.H., Jensen M.K., Curhan G.C., Pasquale L.R., Ridker P.M., Hunter D.J., Willett W.C., Rimm E.B. (2012). Sugar-sweetened beverages and genetic risk of obesity. N. Engl. J. Med..

[11] Gkirtzimanaki K., Gkouskou K.K., Oleksiewicz U., Nikolaidis G., Vyrla D., Liontos M., Pelekanou V., Kanellis D.C., Evangelou K., Stathopoulos E.N. (2013). TPL2 kinase is a suppressor of lung carcinogenesis. Proc. Natl. Acad. Sci. USA.

[12] Vyrla D., Nikolaidis G., Oakley F., Perugorria M.J., Tsichlis P.N., Mann D.A., Eliopoulos A.G. (2016). TPL2 Kinase Is a Crucial Signaling Factor and Mediator of NKT Effector Cytokine Expression in Immune-Mediated Liver Injury. J. Immunol..

[13] Goldsmith D.R., Rapaport M.H., Miller B.J. (2016). A meta-analysis of blood cytokine network alterations in psychiatric patients: Comparisons between schizophrenia, bipolar disorder and depression. Mol. Psychiatry.

[14] Schleifer S.J., Keller S.E., Bartlett J.A. (1999). Depression and immunity: Clinical factors and therapeutic course. Psychiatry Res..

[15] Kendler K.S., Karkowski L.M., Prescott C.A. (1999). Causal relationship between stressful life events and the onset of major depression. Am. J. Psychiatry.

[16] Pariante C.M., Lightman S.L. (2008). The HPA axis in major depression: Classical theories and new developments. Trends Neurosci..

[17] Capellino S., Claus M., Watzl C. (2020). Regulation of natural killer cell activity by glucocorticoids, serotonin, dopamine, and epinephrine. Cell Mol. Immunol..

[18] Ran G.H., Lin Y.Q., Tian L., Zhang T., Yan D.M., Yu J.H., Deng Y.C. (2022). Natural killer cell homing and trafficking in tissues and tumors: From biology to application. Signal Transduct. Target Ther..

[19] Abe S., Kagao M., Asahi T., Kato R., Tani-Ichi S., Shimba A., Ishibashi R., Miyachi H., Kitano S., Miyazaki M. (2025). The transcription factor RORalpha is required for the development of type 1 innate lymphoid cells in adult bone marrow. J. Immunol..

[20] Hellstrand K., Hermodsson S. (1993). Serotonergic 5-HT1A receptors regulate a cell contact-mediated interaction between natural killer cells and monocytes. Scand. J. Immunol..

[21] Sharma R., Das A. (2014). Organ-specific phenotypic and functional features of NK cells in humans. Immunol. Res..

[22] Chaves Filho A.J.M., Lima C.N.C., Vasconcelos S.M.M., de Lucena D.F., Maes M., Macedo D. (2018). IDO chronic immune activation and tryptophan metabolic pathway: A potential pathophysiological link between depression and obesity. Prog. Neuropsychopharmacol. Biol. Psychiatry.

[23] Rodriguez-Mogeda C., van Ansenwoude C.M.J., van der Molen L., Strijbis E.M.M., Mebius R.E., de Vries H.E. (2024). The role of CD56(bright) NK cells in neurodegenerative disorders. J. Neuroinflammation.

[24] Huebner L., Engeli S., Wrann C.D., Goudeva L., Laue T., Kielstein H. (2013). Human NK cell subset functions are differentially affected by adipokines. PLoS ONE.

[25] Kappelmann N., Czamara D., Rost N., Moser S., Schmoll V., Trastulla L., Stochl J., Lucae S., Binder E.B., Khandaker G.M. (2021). Polygenic risk for immuno-metabolic markers and specific depressive symptoms: A multi-sample network analysis study. Brain Behav. Immun..

[26] Khandaker G.M., Pearson R.M., Zammit S., Lewis G., Jones P.B. (2014). Association of serum interleukin 6 and C-reactive protein in childhood with depression and psychosis in young adult life: A population-based longitudinal study. JAMA Psychiatry.

