# Supplementary material for: Genetic Risk for Depression Associates with Circulating Immunoregulatory Natural Killer Cells Independent of BMI: An Exploratory Immunophenotyping Study
Source: Cells. 2026 Jun 29;15(13):1179. doi: 10.3390/cells15131179 (PMC13359601; doi:10.3390/cells15131179)
Supplement: Supplementary file 1 [file cells-15-01179-s001.zip › cells-4358567-supplementary.pdf]

Supplementary Table S1. Descriptive statistics and Spearman correlations with the Depression-GRS for all 116 flow cytometry-derived immune parameters

Values are median (IQR). OW = overweight. * = nominally significant (p<0.05, uncorrected) in the total cohort.

Immune Parameter	Total cohort (n=53)			Lean (n=12)			Overweight (n=9)			Obese (n=32)			Depression-GRS correlation			Sig.
Parameter	n	Median	IQR	n	Median	IQR	n	Median	IQR	n	Median	IQR	ρ	p-value	n	*
**%CD56brightCD16- to DAPI-**	**41**	**0.0024**	**0.0013 – 0.0029**	**11**	**0.0022**	**0.0017 – 0.0025**	**8**	**0.0022**	**0.0012 – 0.0029**	**22**	**0.0025**	**0.0014 – 0.0031**	**0,444**	**0,0036**	**41**	*
**%CD56brightCD16- to SS/FS**	**41**	**0.0025**	**0.0014 – 0.0032**	**11**	**0.0023**	**0.0018 – 0.0027**	**8**	**0.0024**	**0.0012 – 0.0031**	**22**	**0.0027**	**0.0016 – 0.0033**	**0,4318**	**0,0048**	**41**	*
**%CD45RA-CD45RO+ to DAPI-**	**52**	**0.1106**	**0.0864 – 0.1470**	**12**	**0.0951**	**0.0855 – 0.1066**	**9**	**0.0825**	**0.0706 – 0.1441**	**31**	**0.1198**	**0.0984 – 0.1541**	**0,3314**	**0,0164**	**52**	*
**Not NK (CD56-) to CD45+**	**41**	**0.8920**	**0.8440 – 0.9180**	**11**	**0.8750**	**0.8450 – 0.8990**	**8**	**0.9070**	**0.8572 – 0.9240**	**22**	**0.8905**	**0.8460 – 0.9203**	**-0,3533**	**0,0235**	**41**	*
**%CD45R0+CCR7- to DAPI-**	**52**	**0.1356**	**0.1021 – 0.1794**	**12**	**0.1150**	**0.1004 – 0.1516**	**9**	**0.0946**	**0.0743 – 0.1356**	**31**	**0.1677**	**0.1094 – 0.1985**	**0,3081**	**0,0263**	**52**	*
**%CD56brightCD16- to CD45+**	**41**	**0.0029**	**0.0021 – 0.0039**	**11**	**0.0025**	**0.0021 – 0.0031**	**8**	**0.0032**	**0.0023 – 0.0040**	**22**	**0.0032**	**0.0019 – 0.0039**	**0,342**	**0,0286**	**41**	*
**% SS/FS**	**52**	**0.9425**	**0.9093 – 0.9645**	**12**	**0.9270**	**0.9030 – 0.9532**	**9**	**0.9240**	**0.8880 – 0.9740**	**31**	**0.9470**	**0.9235 – 0.9720**	**0,2861**	**0,0397**	**52**	*
**%CD56+ to DAPI-**	**41**	**0.0680**	**0.0468 – 0.0971**	**11**	**0.0723**	**0.0566 – 0.1004**	**8**	**0.0593**	**0.0365 – 0.0808**	**22**	**0.0643**	**0.0516 – 0.0953**	**0,3214**	**0,0405**	**41**	*
**%CD56+ to CD45+**	**41**	**0.0850**	**0.0630 – 0.1250**	**11**	**0.0950**	**0.0655 – 0.1310**	**8**	**0.0700**	**0.0535 – 0.0927**	**22**	**0.0930**	**0.0695 – 0.1143**	**0,3204**	**0,0411**	**41**	*
**%CD45RA-CD45RO+ to SS/FS**	**52**	**0.1203**	**0.0959 – 0.1541**	**12**	**0.1014**	**0.0929 – 0.1290**	**9**	**0.0898**	**0.0724 – 0.1513**	**31**	**0.1384**	**0.1096 – 0.1690**	**0,2821**	**0,0427**	**52**	*
**%CD45R0+CXCR3+ to DAPI-**	**52**	**0.0401**	**0.0236 – 0.0594**	**12**	**0.0410**	**0.0297 – 0.0465**	**9**	**0.0233**	**0.0208 – 0.0500**	**31**	**0.0450**	**0.0242 – 0.0682**	**0,275**	**0,0485**	**52**	*
**%CD45RA+CCR7+ to DAPI-**	**52**	**0.0166**	**0.0092 – 0.0277**	**12**	**0.0175**	**0.0122 – 0.0239**	**9**	**0.0093**	**0.0089 – 0.0112**	**31**	**0.0190**	**0.0114 – 0.0280**	**0,2738**	**0,0495**	**52**	*
%CD56dimCD16- to DAPI-	41	0.0119	0.0082 – 0.0179	11	0.0109	0.0088 – 0.0152	8	0.0145	0.0100 – 0.0240	22	0.0124	0.0082 – 0.0190	0,3047	0,0527	41	
%CD14+CD16+ to SS/FS	47	0.0124	0.0056 – 0.0185	11	0.0115	0.0096 – 0.0187	9	0.0054	0.0050 – 0.0124	27	0.0150	0.0064 – 0.0191	0,2783	0,0582	47	
%Tregs to CD4	52	0.0670	0.0578 – 0.0803	12	0.0755	0.0648 – 0.0850	9	0.0800	0.0660 – 0.0970	31	0.0650	0.0535 – 0.0715	-0,2574	0,0655	52	
%CD14+CD16+ to DAPI-	47	0.0122	0.0052 – 0.0179	11	0.0106	0.0091 – 0.0171	9	0.0050	0.0048 – 0.0122	27	0.0145	0.0060 – 0.0188	0,2687	0,0678	47	
%CD45R0+CCR7- to SS/FS	52	0.1437	0.1134 – 0.1881	12	0.1254	0.1082 – 0.1627	9	0.1066	0.0763 – 0.1422	31	0.1739	0.1362 – 0.2099	0,2525	0,071	52	
%CD56+ to SS/FS	41	0.0717	0.0478 – 0.1074	11	0.0767	0.0597 – 0.1111	8	0.0624	0.0411 – 0.0872	22	0.0685	0.0568 – 0.1009	0,2756	0,0811	41	
%CD45R0+CXCR3+ to SS/FS	52	0.0457	0.0251 – 0.0661	12	0.0459	0.0320 – 0.0513	9	0.0266	0.0213 – 0.0547	31	0.0488	0.0270 – 0.0752	0,2438	0,0816	52	
%CD14+CD16- to Not NK	41	0.1167	0.0766 – 0.1518	11	0.1212	0.0774 – 0.1826	8	0.0978	0.0828 – 0.1174	22	0.1206	0.0742 – 0.1502	0,2718	0,0856	41	
%CD56dimCD16- to SS/FS	41	0.0130	0.0091 – 0.0192	11	0.0113	0.0094 – 0.0170	8	0.0160	0.0109 – 0.0247	22	0.0132	0.0094 – 0.0199	0,2686	0,0895	41	
%CD14+CD16+ to Not Neu	47	0.0160	0.0100 – 0.0220	11	0.0160	0.0130 – 0.0305	9	0.0130	0.0070 – 0.0140	27	0.0190	0.0110 – 0.0215	0,2426	0,1004	47	
%CD14+CD16+ to CD45+	47	0.0157	0.0085 – 0.0211	11	0.0136	0.0102 – 0.0246	9	0.0117	0.0063 – 0.0128	27	0.0161	0.0099 – 0.0211	0,2396	0,1048	47	
%CD45RA+CCR7+ to SS/FS	52	0.0191	0.0096 – 0.0284	12	0.0199	0.0128 – 0.0255	9	0.0106	0.0089 – 0.0120	31	0.0258	0.0125 – 0.0298	0,2246	0,1094	52	
%CD14+CD16- to SS/FS	47	0.0960	0.0556 – 0.1239	11	0.0960	0.0556 – 0.1479	9	0.0636	0.0633 – 0.0934	27	0.1003	0.0521 – 0.1205	0,2331	0,1148	47	
%CD14+CD16- to DAPI-	47	0.0849	0.0513 – 0.1181	11	0.0930	0.0513 – 0.1398	9	0.0609	0.0589 – 0.0849	27	0.0960	0.0450 – 0.1131	0,2326	0,1157	47	
%CD4+ to DAPI-	52	0.2703	0.2154 – 0.3282	12	0.2307	0.1892 – 0.2613	9	0.2827	0.2616 – 0.3079	31	0.3011	0.2265 – 0.3521	0,2196	0,1178	52	
%CD4+ to CD3	52	0.6210	0.5585 – 0.6683	12	0.5605	0.5022 – 0.6212	9	0.6090	0.5650 – 0.6320	31	0.6340	0.5835 – 0.6800	0,2184	0,1198	52	
%CD45R0+CCR7+ to DAPI-	52	0.0033	0.0016 – 0.0058	12	0.0033	0.0017 – 0.0039	9	0.0031	0.0015 – 0.0049	31	0.0042	0.0019 – 0.0073	0,2177	0,121	52	
%CD45RA+CCR7- to CD45RA+	52	0.7760	0.6505 – 0.8928	12	0.7720	0.6020 – 0.8965	9	0.9100	0.8170 – 0.9320	31	0.7500	0.6200 – 0.8530	-0,2144	0,1269	52	
Not Neutrophils to CD45+	47	0.8732	0.8377 – 0.9257	11	0.8396	0.8106 – 0.8531	9	0.9082	0.8587 – 0.9287	27	0.8795	0.8489 – 0.9515	-0,2235	0,131	47	
%CD45RA+CCR7+ to CD45RA+	52	0.2180	0.1072 – 0.3495	12	0.2250	0.1035 – 0.3980	9	0.0900	0.0610 – 0.1810	31	0.2380	0.1470 – 0.3800	0,2079	0,1392	52	
%CD56+CD16+ to SS/FS	41	0.0524	0.0276 – 0.0743	11	0.0618	0.0404 – 0.0759	8	0.0353	0.0214 – 0.0548	22	0.0516	0.0320 – 0.0715	0,2276	0,1523	41	
%CD45RA+CCR7+ to CD3	52	0.0453	0.0208 – 0.0590	12	0.0453	0.0257 – 0.0729	9	0.0199	0.0181 – 0.0377	31	0.0487	0.0232 – 0.0670	0,1986	0,1581	52	
%CD56+CD16+ to CD45+	41	0.0596	0.0426 – 0.0821	11	0.0766	0.0448 – 0.0905	8	0.0391	0.0288 – 0.0583	22	0.0593	0.0446 – 0.0798	0,2243	0,1585	41	
%CD56+CD16+ to DAPI-	41	0.0493	0.0258 – 0.0667	11	0.0577	0.0388 – 0.0701	8	0.0336	0.0189 – 0.0507	22	0.0474	0.0294 – 0.0651	0,2234	0,1604	41	
%CD14+CD16- to CD45+	47	0.1044	0.0702 – 0.1355	11	0.1093	0.0659 – 0.1607	9	0.0892	0.0737 – 0.1003	27	0.1107	0.0772 – 0.1345	0,2061	0,1646	47	
%CD45R0+CCR7+ to SS/FS	52	0.0035	0.0017 – 0.0062	12	0.0035	0.0019 – 0.0044	9	0.0031	0.0015 – 0.0055	31	0.0042	0.0020 – 0.0078	0,1931	0,1702	52	
%CD14+CD16+ to Not NK	41	0.0139	0.0093 – 0.0207	11	0.0152	0.0128 – 0.0288	8	0.0111	0.0069 – 0.0139	22	0.0175	0.0094 – 0.0201	0,2121	0,1832	41	
%CD45+ to DAPI-	47	0.8463	0.7326 – 0.8949	11	0.8503	0.7659 – 0.8857	9	0.8463	0.7342 – 0.8838	27	0.8148	0.7211 – 0.9006	0,1969	0,1847	47	
%CD45R0+CCR7- to CD3	52	0.2320	0.2033 – 0.3060	12	0.2168	0.2046 – 0.2963	9	0.1886	0.1394 – 0.2383	31	0.2664	0.2167 – 0.3137	0,1864	0,1857	52	
%CD45RA+CD45RO- to CD4	52	0.3275	0.2278 – 0.4062	12	0.3985	0.2655 – 0.4120	9	0.3830	0.2470 – 0.3920	31	0.2890	0.2205 – 0.3710	-0,1848	0,1897	52	

%CD8+ to CD3	50	0.2560	0.2095 – 0.3113	12	0.3020	0.2510 – 0.3393	9	0.2730	0.1950 – 0.3140	29	0.2450	0.2080 – 0.3020	-0,1837	0,2015	50	
%CD14+CD16- to Not Neu	47	0.1200	0.0835 – 0.1560	11	0.1280	0.0835 – 0.1845	9	0.1010	0.0850 – 0.1260	27	0.1220	0.0805 – 0.1500	0,1819	0,221	47	
%CD3+ to DAPI-	52	0.4524	0.4022 – 0.5260	12	0.4183	0.3757 – 0.4388	9	0.4547	0.4454 – 0.5463	31	0.4831	0.3888 – 0.5261	0,1674	0,2354	52	
%CD4+ to SS/FS	52	0.3096	0.2414 – 0.3614	12	0.2450	0.2137 – 0.2762	9	0.3162	0.2893 – 0.3205	31	0.3364	0.2534 – 0.3655	0,1666	0,2379	52	
%CD14-CD16- to DAPI-	47	0.5045	0.4166 – 0.5603	11	0.4868	0.3668 – 0.5148	9	0.5206	0.4639 – 0.5734	27	0.5385	0.4250 – 0.5626	0,1751	0,2391	47	
%CD14-CD16- to Not Neu	47	0.7480	0.6910 – 0.7805	11	0.7400	0.6845 – 0.7690	9	0.7650	0.7360 – 0.7840	27	0.7480	0.6910 – 0.7855	-0,1734	0,2437	47	
%CD45RA+CCR7+ to CD4	52	0.0719	0.0337 – 0.1031	12	0.0853	0.0430 – 0.1239	9	0.0347	0.0279 – 0.0666	31	0.0739	0.0403 – 0.0979	0,1632	0,2478	52	
%CD45RA+CXCR3+ to DAPI-	52	0.0022	0.0014 – 0.0036	12	0.0021	0.0013 – 0.0041	9	0.0023	0.0015 – 0.0052	31	0.0023	0.0015 – 0.0035	0,1571	0,2659	52	
%CD14lowCD16+ to DAPI-	44	0.0065	0.0042 – 0.0090	10	0.0060	0.0039 – 0.0077	9	0.0057	0.0021 – 0.0072	25	0.0066	0.0050 – 0.0097	0,1679	0,276	44	
% SS/FS	47	0.9490	0.9150 – 0.9640	11	0.9410	0.9230 – 0.9505	9	0.9270	0.9090 – 0.9610	27	0.9580	0.9090 – 0.9665	0,1613	0,2787	47	
%CD14-CD16-HLADR+ to Not Neu	47	0.0894	0.0701 – 0.1071	11	0.0924	0.0658 – 0.1119	9	0.0723	0.0567 – 0.0828	27	0.0930	0.0712 – 0.1036	0,1607	0,2806	47	
%CD15+CD16+ to SS/FS	47	0.0140	0.0034 – 0.0338	11	0.0412	0.0082 – 0.0441	9	0.0039	0.0033 – 0.0153	27	0.0153	0.0034 – 0.0279	0,1576	0,2902	47	
%CD14lowCD16+ to SS/FS	44	0.0069	0.0046 – 0.0096	10	0.0063	0.0041 – 0.0088	9	0.0062	0.0022 – 0.0076	25	0.0070	0.0055 – 0.0105	0,1617	0,2944	44	
%CD45R0+CXCR3+ to CD3	52	0.1048	0.0622 – 0.1356	12	0.1063	0.0697 – 0.1162	9	0.0557	0.0414 – 0.1324	31	0.1030	0.0645 – 0.1458	0,1466	0,2997	52	
%Tregs to CD3	50	0.0416	0.0349 – 0.0496	12	0.0417	0.0383 – 0.0494	9	0.0494	0.0358 – 0.0603	29	0.0387	0.0346 – 0.0468	-0,1473	0,3072	50	
%CD14-CD16- to Not NK	41	0.7311	0.6877 – 0.7754	11	0.6977	0.6724 – 0.7213	8	0.7621	0.7231 – 0.7839	22	0.7383	0.6820 – 0.7831	-0,1582	0,3232	41	
%CD15+CD16+ to DAPI-	47	0.0134	0.0033 – 0.0312	11	0.0382	0.0077 – 0.0409	9	0.0036	0.0032 – 0.0141	27	0.0146	0.0033 – 0.0269	0,1468	0,325	47	
%CD45RA-CD45RO+ to CD3	52	0.2484	0.2137 – 0.3190	12	0.2240	0.2119 – 0.3156	9	0.2417	0.1782 – 0.2696	31	0.2730	0.2308 – 0.3335	0,1377	0,3304	52	
%CD15+CD16+ to CD45+	47	0.0160	0.0039 – 0.0411	11	0.0469	0.0087 – 0.0505	9	0.0054	0.0037 – 0.0160	27	0.0176	0.0050 – 0.0340	0,1417	0,342	47	
%CD14-CD16-HLADR+ to CD14-CD16-	47	0.1270	0.0985 – 0.1420	11	0.1360	0.0995 – 0.1645	9	0.0990	0.0730 – 0.1300	27	0.1270	0.1020 – 0.1420	0,141	0,3446	47	
%CD45+ to SS/FS	47	0.8810	0.8080 – 0.9360	11	0.8780	0.8250 – 0.9265	9	0.9290	0.8090 – 0.9580	27	0.8810	0.7980 – 0.9360	0,1368	0,3591	47	
%CD15+CD16+ to CD56-	41	0.0160	0.0040 – 0.0490	11	0.0520	0.0115 – 0.0580	8	0.0055	0.0040 – 0.0132	22	0.0190	0.0033 – 0.0447	0,1403	0,3817	41	
%CD56brightCD16- to CD56+	41	0.0310	0.0200 – 0.0570	11	0.0300	0.0185 – 0.0455	8	0.0520	0.0220 – 0.0743	22	0.0305	0.0245 – 0.0520	0,1341	0,4033	41	
%CD45RA+CXCR3+ to SS/FS	52	0.0024	0.0016 – 0.0038	12	0.0023	0.0015 – 0.0043	9	0.0023	0.0015 – 0.0055	31	0.0025	0.0016 – 0.0038	0,1177	0,4061	52	
%CD14lowCD16+ to CD45+	44	0.0078	0.0059 – 0.0113	10	0.0067	0.0049 – 0.0095	9	0.0067	0.0054 – 0.0091	25	0.0093	0.0062 – 0.0121	0,1274	0,4101	44	
%CD45R0+CCR7+ to CD3	52	0.0075	0.0036 – 0.0134	12	0.0071	0.0048 – 0.0089	9	0.0054	0.0036 – 0.0108	31	0.0100	0.0035 – 0.0165	0,1161	0,4123	52	
%CD14-CD16- to CD45+	47	0.6306	0.5900 – 0.6987	11	0.5891	0.5464 – 0.6369	9	0.7057	0.6008 – 0.7314	27	0.6436	0.5983 – 0.6908	-0,1202	0,4211	47	
%CD14lowCD16+ to Not NK	38	0.0080	0.0061 – 0.0125	10	0.0084	0.0057 – 0.0112	8	0.0074	0.0052 – 0.0102	20	0.0089	0.0068 – 0.0134	0,1344	0,4212	38	
%Tregs to DAPI-	52	0.0189	0.0164 – 0.0229	12	0.0173	0.0152 – 0.0205	9	0.0245	0.0182 – 0.0274	31	0.0191	0.0163 – 0.0223	0,1124	0,4276	52	
%CD56+CD16+ to CD56+	41	0.6650	0.5680 – 0.7630	11	0.6820	0.6195 – 0.7915	8	0.5665	0.5188 – 0.5958	22	0.6905	0.5955 – 0.7840	0,1272	0,4281	41	
%CD14-CD16+ to Not NK	41	0.0236	0.0130 – 0.0419	11	0.0236	0.0193 – 0.0365	8	0.0448	0.0214 – 0.0691	22	0.0202	0.0116 – 0.0370	-0,1255	0,4342	41	
%CD56dimCD16- to CD56+	41	0.1980	0.1410 – 0.3110	11	0.1740	0.1265 – 0.2195	8	0.2860	0.2347 – 0.3390	22	0.1930	0.1395 – 0.2535	-0,1158	0,4711	41	
%CD56dimCD16- to CD45+	41	0.0159	0.0122 – 0.0239	11	0.0128	0.0111 – 0.0237	8	0.0187	0.0166 – 0.0264	22	0.0150	0.0122 – 0.0273	0,1156	0,4717	41	
Not Neutrophils to DAPI-	44	0.7098	0.6295 – 0.7727	10	0.6949	0.6228 – 0.7350	9	0.7073	0.6466 – 0.7905	25	0.7470	0.6599 – 0.7941	0,1041	0,5013	44	
%CD14lowCD16+ to Not Neu	44	0.0100	0.0067 – 0.0130	10	0.0085	0.0060 – 0.0118	9	0.0080	0.0060 – 0.0110	25	0.0100	0.0070 – 0.0150	0,1033	0,5045	44	
%CD45RA+CD45RO- to CD3	52	0.1922	0.1390 – 0.2308	12	0.2021	0.1425 – 0.2258	9	0.2089	0.1551 – 0.2215	31	0.1825	0.1333 – 0.2414	-0,0899	0,5264	52	
%CD3+ to SS/FS	52	0.4980	0.4367 – 0.5608	12	0.4415	0.4130 – 0.4680	9	0.5120	0.4920 – 0.5780	31	0.5330	0.4495 – 0.5615	0,0817	0,565	52	
%CD45R0+CCR7+ to CD4	52	0.0118	0.0065 – 0.0215	12	0.0119	0.0096 – 0.0157	9	0.0099	0.0057 – 0.0186	31	0.0130	0.0063 – 0.0270	0,0808	0,5692	52	
%CD14-CD16+ to DAPI-	47	0.0184	0.0106 – 0.0326	11	0.0176	0.0121 – 0.0210	9	0.0290	0.0142 – 0.0387	27	0.0190	0.0081 – 0.0337	0,0825	0,5816	47	
Not Neutrophils to SS/FS	44	0.7701	0.6759 – 0.8200	10	0.7341	0.6727 – 0.7707	9	0.7701	0.7347 – 0.8226	25	0.7883	0.6919 – 0.8439	0,0799	0,6061	44	
Not Neutrophils to CD56-	38	0.9800	0.9502 – 0.9948	10	0.9490	0.9415 – 0.9840	8	0.9945	0.9865 – 0.9950	20	0.9700	0.9525 – 0.9920	-0,0835	0,6184	38	
%CD45RA+CXCR3+ to CD4	52	0.0094	0.0062 – 0.0138	12	0.0107	0.0087 – 0.0151	9	0.0106	0.0067 – 0.0157	31	0.0088	0.0062 – 0.0126	-0,0634	0,6553	52	
%CD45RA+CCR7- to DAPI-	52	0.0979	0.0599 – 0.1398	12	0.0761	0.0475 – 0.0922	9	0.1296	0.1149 – 0.1419	31	0.1000	0.0599 – 0.1444	0,0634	0,6555	52	
%CD14-CD16+ to SS/FS	47	0.0198	0.0118 – 0.0339	11	0.0186	0.0143 – 0.0234	9	0.0319	0.0162 – 0.0403	27	0.0202	0.0096 – 0.0344	0,0657	0,6606	47	
%CD14-CD16- to SS/FS	47	0.5648	0.4462 – 0.5809	11	0.5060	0.3895 – 0.5483	9	0.5727	0.5155 – 0.5860	27	0.5658	0.4684 – 0.6024	0,0571	0,7029	47	
%CD45RA+CCR7- to CD4	52	0.4683	0.3739 – 0.5531	12	0.3645	0.2902 – 0.4831	9	0.5043	0.4689 – 0.5853	31	0.4712	0.3792 – 0.5549	-0,0534	0,7071	52	
%CD14-CD16-HLADR+ to Not NK	41	0.0907	0.0689 – 0.1117	11	0.1002	0.0697 – 0.1281	8	0.0676	0.0563 – 0.0839	22	0.0930	0.0711 – 0.1118	0,0598	0,7105	41	
%CD45R0+CXCR3+ to CD4	52	0.1704	0.1114 – 0.2273	12	0.1706	0.1504 – 0.2009	9	0.1018	0.0794 – 0.2268	31	0.1707	0.1110 – 0.2308	0,051	0,7197	52	
#of PBMC	49	24200000.	19000000.0000 –	11	16800000.	15700000.0000 –	9	22000000.	19000000.0000 –	29	27000000.	20800000.0000 –	0,0501	0,7326	49	
%CD14-CD16-HLADR+ to DAPI-	47	0.0631	0.0466 – 0.0790	11	0.0677	0.0472 – 0.0768	9	0.0574	0.0367 – 0.0677	27	0.0632	0.0535 – 0.0846	0,0506	0,7356	47	
%CD8+ to SS/FS	52	0.1266	0.0932 – 0.1506	12	0.1344	0.1057 – 0.1452	9	0.1141	0.0921 – 0.1602	31	0.1252	0.0928 – 0.1547	-0,0459	0,7469	52	

Not NK (CD56-) to SS/FS	41	0.7553	0.6473 – 0.8099	11	0.7553	0.6802 – 0.8010	8	0.7691	0.6885 – 0.7932	22	0.7339	0.6472 – 0.8213	0,0512	0,7504	41	
Not NK (CD56-) to DAPI-	41	0.6933	0.5811 – 0.7682	11	0.6972	0.6366 – 0.7648	8	0.7093	0.6153 – 0.7361	22	0.6822	0.5761 – 0.7734	0,049	0,7607	41	
%Tregs to SS/FS	52	0.0198	0.0170 – 0.0236	12	0.0176	0.0154 – 0.0207	9	0.0247	0.0192 – 0.0276	31	0.0198	0.0173 – 0.0232	0,0425	0,7649	52	
%CD45RA+CXCR3+ to CD45RA+	52	0.0320	0.0220 – 0.0430	12	0.0300	0.0235 – 0.0457	9	0.0410	0.0150 – 0.0440	31	0.0300	0.0230 – 0.0425	0,0376	0,791	52	
%CD45RA+CD45RO- to DAPI-	52	0.0818	0.0554 – 0.1128	12	0.0836	0.0599 – 0.0996	9	0.1007	0.0622 – 0.1165	31	0.0739	0.0553 – 0.1176	0,0334	0,814	52	
%CD45RA+CD45RO+ to DAPI-	52	0.0040	0.0028 – 0.0085	12	0.0037	0.0023 – 0.0054	9	0.0061	0.0031 – 0.0085	31	0.0040	0.0028 – 0.0086	0,033	0,8163	52	
%CD45RA-CD45RO+ to CD4	52	0.4325	0.3728 – 0.5030	12	0.4390	0.3840 – 0.4920	9	0.4000	0.2820 – 0.4240	31	0.4550	0.3840 – 0.5060	-0,0242	0,865	52	
%CD45R0+CCR7+ to CD45RO+	52	0.0320	0.0187 – 0.0540	12	0.0315	0.0212 – 0.0400	9	0.0340	0.0210 – 0.0540	31	0.0300	0.0140 – 0.0640	0,019	0,8934	52	
%CD45RA+CCR7- to SS/FS	52	0.1120	0.0645 – 0.1504	12	0.0798	0.0532 – 0.0959	9	0.1417	0.1257 – 0.1536	31	0.1128	0.0641 – 0.1633	0,0175	0,9022	52	
%CD14-CD16-HLADR+ to SS/FS	47	0.0659	0.0495 – 0.0863	11	0.0713	0.0511 – 0.0829	9	0.0621	0.0417 – 0.0745	27	0.0659	0.0560 – 0.0877	0,0176	0,9066	47	
%CD45RA+CXCR3+ to CD3	52	0.0059	0.0039 – 0.0090	12	0.0056	0.0047 – 0.0091	9	0.0067	0.0040 – 0.0094	31	0.0059	0.0036 – 0.0077	0,0154	0,9136	52	
%CD45R0+CCR7- to CD45RO+	52	0.9610	0.9340 – 0.9775	12	0.9610	0.9517 – 0.9742	9	0.9510	0.9410 – 0.9790	31	0.9590	0.9310 – 0.9825	0,0135	0,9246	52	
%CD45R0+CCR7- to CD4	52	0.4162	0.3400 – 0.4776	12	0.4264	0.3740 – 0.4739	9	0.3338	0.2634 – 0.4181	31	0.4190	0.3537 – 0.4813	0,0112	0,937	52	
%CD45RA+CD45RO+ to CD3	52	0.0100	0.0070 – 0.0166	12	0.0088	0.0055 – 0.0131	9	0.0139	0.0057 – 0.0186	31	0.0103	0.0072 – 0.0166	0,0099	0,9447	52	
%CD14-CD16+ to Not Neu	47	0.0300	0.0180 – 0.0565	11	0.0250	0.0205 – 0.0370	9	0.0490	0.0220 – 0.0850	27	0.0280	0.0130 – 0.0565	0,0091	0,9518	47	
%CD45RA+CD45RO+ to SS/FS	52	0.0045	0.0032 – 0.0090	12	0.0039	0.0024 – 0.0057	9	0.0070	0.0032 – 0.0095	31	0.0054	0.0032 – 0.0090	-0,0075	0,9578	52	
%CD45RA+CD45RO+ to CD4	52	0.0160	0.0110 – 0.0270	12	0.0160	0.0097 – 0.0195	9	0.0220	0.0110 – 0.0320	31	0.0160	0.0120 – 0.0265	-0,0046	0,9741	52	
%CD14-CD16+ to CD45+	47	0.0254	0.0145 – 0.0423	11	0.0206	0.0174 – 0.0309	9	0.0375	0.0200 – 0.0777	27	0.0254	0.0111 – 0.0528	-0,0046	0,9754	47	
%CD14-CD16-HLADR+ to CD45+	47	0.0765	0.0603 – 0.0958	11	0.0765	0.0607 – 0.1118	9	0.0661	0.0515 – 0.0800	27	0.0829	0.0639 – 0.0967	0,0043	0,9769	47	
%CD45R0+CXCR3+ to CD45R0+	52	0.3920	0.2975 – 0.4882	12	0.4400	0.3260 – 0.4852	9	0.3800	0.2960 – 0.5350	31	0.3440	0.2705 – 0.4775	-0,0036	0,9797	52	
%CD8+ to DAPI-	52	0.1206	0.0889 – 0.1459	12	0.1335	0.1028 – 0.1442	9	0.1100	0.0893 – 0.1594	31	0.1147	0.0890 – 0.1462	-0,0031	0,9825	52	
%CD45RA+CD45RO- to SS/FS	52	0.0883	0.0623 – 0.1268	12	0.0892	0.0649 – 0.1047	9	0.1033	0.0673 – 0.1224	31	0.0828	0.0612 – 0.1291	0,0014	0,9921	52	
%CD45RA+CCR7- to CD3	52	0.2339	0.1543 – 0.2875	12	0.1592	0.1241 – 0.2111	9	0.2602	0.2442 – 0.2915	31	0.2367	0.1623 – 0.2903	0,0012	0,9935	52	

Suppl. Table S2: Biological pathway categories identified among the 56 genes represented in the Depression-GRS. Genes with direct relevance to NK cell biology are marked in bold.

Pathway	Key genes
Serotonin/monoamine neurotransmission	*TPH1*, ***HTR1A***, *HTR1B, HTR2A, SLC6A15*, ***COMT***
HPA axis / stress response	***CRHR1, CRHR2, CRHBP***
Neurotrophin / synaptic plasticity	*BDNF, HOMER1, PCLO, SYT4*
Glutamate receptor signalling	*GRM7, GRM8*
G-protein / intracellular signalling	*GNAI3, GNB3, DVL3, SHC4*
Circadian rhythm / nuclear receptors	***RORA***
Metabolic / energy regulation	*FTO, MTHFR, SIRT1, EHHADH, APOE*
Cell adhesion / migration / ECM	***ITGB1***, *NRP1, CDH13, CDH18, VCAN, HAPLN1, SDC1*
Interferon / immune signalling	***SLFN12L, SLFN14,*** *PARP11, PARP15*
PI3K-Akt Signaling Pathway	*BDNF, GNB3*, ***ITGB1***, *GNAI3*
Vesicular / lysosomal function	*ATP6V1B2, SYT4*
